# Clonal dissemination of *Klebsiella pneumoniae* resistant to cefiderocol, ceftazidime/avibactam, meropenem/vaborbactam and imipenem/relebactam co-producing KPC and OXA-181 carbapenemase

**DOI:** 10.1093/jacamr/dlad099

**Published:** 2023-08-17

**Authors:** Federica Bovo, Stefano Amadesi, Marta Palombo, Tiziana Lazzarotto, Simone Ambretti, Paolo Gaibani

**Affiliations:** Division of Microbiology, IRCCS Azienda Ospedaliero-Universitaria di Bologna, S. Orsola-Malpighi University Hospital, via G. Massarenti 9–40138, Bologna, Italy; Division of Microbiology, IRCCS Azienda Ospedaliero-Universitaria di Bologna, S. Orsola-Malpighi University Hospital, via G. Massarenti 9–40138, Bologna, Italy; Division of Microbiology, IRCCS Azienda Ospedaliero-Universitaria di Bologna, S. Orsola-Malpighi University Hospital, via G. Massarenti 9–40138, Bologna, Italy; Division of Microbiology, IRCCS Azienda Ospedaliero-Universitaria di Bologna, S. Orsola-Malpighi University Hospital, via G. Massarenti 9–40138, Bologna, Italy; Department of Medical and Surgical Sciences, University of Bologna, Bologna, Italy; Division of Microbiology, IRCCS Azienda Ospedaliero-Universitaria di Bologna, S. Orsola-Malpighi University Hospital, via G. Massarenti 9–40138, Bologna, Italy; Department of Medical and Surgical Sciences, University of Bologna, Bologna, Italy; Division of Microbiology, IRCCS Azienda Ospedaliero-Universitaria di Bologna, S. Orsola-Malpighi University Hospital, via G. Massarenti 9–40138, Bologna, Italy

## Abstract

**Objectives:**

Herein, we describe the epidemiology of carbapenemase-producing Enterobacterales (CPE) before and during the COVID-19 pandemic. Also, we report the emergence of an outbreak of *Klebsiella pneumoniae* strains co-producing KPC and OXA-181 carbapenemase, resistant to novel β-lactam/β-lactamase inhibitors (βL-βLICs) and cefiderocol.

**Methods:**

CPE were collected during a period of 3 years from 2019 to 2021. Antimicrobial susceptibility testing for novel βL-βLICs and cefiderocol was performed by MIC test strips and microdilution with iron-depleted broth. WGS was performed on 10 selected isolates using the Illumina platform, and resistome analysis was carried out by a web-based pipeline.

**Results:**

Between January 2019 and December 2021, we collected 1430 carbapenemase producers from 957 patients with infections due to CPE. KPC was the most common carbapenemase, followed by VIM, OXA-48 and NDM. During 2021, we identified 78 *K. pneumoniae* co-producing KPC and OXA-181 carbapenemases in 60 patients, resistant to meropenem/vaborbactam and imipenem/relebactam. Resistance to ceftazidime/avibactam and cefiderocol was observed respectively in 7 and 8 out of the 10 sequenced *K. pneumoniae*. Genome analysis showed that all isolates were clonally related, shared a common porin and plasmid content, and carried *bla*_OXA-181_ and *bla*_KPC_ carbapenemases. Specifically, 4 out of 10 isolates carried *bla*_KPC-3_, while 6 harboured mutated *bla*_KPC_. Of note, KPC producers resistant to ceftazidime/avibactam and harbouring mutated *bla*_KPC_ exhibited higher MICs of cefiderocol (median MIC 16 mg/L, IQR 16–16) than strains harbouring WT *bla*_KPC-3_ (cefiderocol 9 mg/L, IQR 1.5–16).

**Conclusions:**

Our results highlight the need for continuous monitoring of CPE to limit widespread MDR pathogens carrying multiple mechanisms conferring resistance to novel antimicrobial molecules.

## Introduction

In the last decade, carbapenemase-producing Enterobacterales (CPE) have become a worldwide health problem,^1^ and in 2017 the WHO included CPE as priority pathogens for which new treatments are needed.^2^

Between 2015 and 2019, new combinations of β-lactam/β-lactamase inhibitors (βL-βLICs) were introduced into clinical practice.^2,3^ However, the emergence of CPE resistant to novel βL-βLICs has been recently reported.^3–5^

Cefiderocol, a novel siderophore cephalosporin, was recently approved by the FDA for the treatment of complicated urinary tract infection (cUTI)^6,7^ and nosocomial pneumonia caused by resistant Gram-negative pathogens.^8^ Despite its promising clinical results, emerging resistant strains have been recently described.^9,10^ Interestingly, it has been recently hypothesized that resistance to ceftazidime/avibactam in KPC-producing Enterobacterales could lead to cross-resistance to cefiderocol.^11,12^ In this study we describe cross-resistance to both βL-βLICs and cefiderocol in *Klebsiella pneumoniae* strains harbouring KPC and OXA-181 carbapenemase, isolated from hospitalized patients during a clonal outbreak occurred in 2021 during the COVID-19 pandemic.

## Materials and methods

### Study setting

Between January 2019 and December 2021, we collected clinical strains isolated at the Operative Unit (OU) of Microbiology. The OU of Microbiology served as the reference centre for microbiological analysis of three different hospitals located in the metropolitan area of Bologna, Emilia-Romagna region, Italy. The three hospitals were: IRCCS Policlinico di Sant’Orsola, a 1420 bed university hospital with an average of 72 000 admissions per year; Maggiore Hospital (MH), a hospital with 870 beds; and Bellaria Hospital (BH), a teaching hospital with 320 beds.

### Bacterial identification

Clinical isolates were identified by MALDI-TOF MS assay (Bruker Daltonics, Germany) and antimicrobial susceptibility testing (AST) was performed using the MicroScan Walkaway system.^13^ MIC results for novel βL-βLICs were confirmed by test strip on regular non-supplemented Mueller–Hinton agar (Liofilchem, Italy) and for cefiderocol using the microdilution reference method utilizing ID-CAMHB.^14^ MICs were interpreted following EUCAST clinical breakpoints v12.0 (https://www.eucast.org/clinical_breakpoints/). Carbapenemase production was detected by the NG-Test CARBA 5 (NG Biotech, France) and confirmed with a molecular assay (Xpert Carba-R, Cepheid, USA).

### Genomic analysis

Ten *K. pneumoniae* isolates positive for both KPC and OXA-48 carbapenemases were selected for genomic analysis in order to generate a representative subset. Samples were collected from individual patients admitted to any of the three mentioned facilities. Serial isolates from the same patient were excluded to ensure genetic diversity.

DNA was extracted from purified cultures of *K. pneumoniae* using the DNeasy Blood & Tissue Kit (QIAGEN, Basel, Switzerland) by following the manufacturer’s instructions, and further cleaned up with AMPure XP magnetic beads (Beckman Coulter). WGS was performed by the Illumina iSeq 100 platform (iSeq Reagent Kit v2, Illumina, San Diego, USA) using iSeq Reagent kit v2 with 2 × 150 paired-end reads after Illumina DNA Prep paired-end library preparation.^15,16^

Read-quality reports were generated using FastQC and *de novo* genome assembly was performed with SPAdes v.3.15.5. ST, plasmid replicon type and antimicrobial resistance genes were investigated as previously described.^15,16^ Sequence variation in genes encoding for the major non-selective porins OmpK35 and OmpK36, and the *bla*_KPC-3_ carbapenemase gene was evaluated by aligning amino acid sequence against reference genes as previously described.^15,16^ Phylogenetic analysis based on core-genome SNPs was performed as previously described.^15,16^ A phylogenetic tree was generated in order to compare the strains included in this study with a set of strains isolated at our facility using strain 101BO (accession no. CCEY01000001) as a reference.

## Results

From January 2019 to December 2021, a total of 56 091 clinical strains isolated were collected at the OU of Microbiology. During the study period, a total of 957 patients were infected by CPE, showing a prevalence of 2.5% (1430/56 091) among clinical specimens. Overall, the yearly proportion of CPE clinical isolates was consistent across the study period (2.27% in 2019; 2.31% in 2020; 3.07% in 2021). Deeper examination of the carbapenemase epidemiology showed that KPC was the most prevalent enzyme (75.4% 1078/1430), followed by OXA-48-like (8.3%; 118/1430), VIM (6.7%; 96/1430), KPC + OXA_48_ (5.4%; 78/1430) and NDM (4.2%; 60/1430). Epidemiology of carbapenemase mechanisms during 2019 to 2021 is reported in Figure S1 (available as Supplementary data at *JAC-AMR* Online). Since 2020, (i.e. the beginning of the COVID-19 pandemic), carbapenemase epidemiology has showed a significant decrease of KPC enzyme and a concomitant increase of OXA48-like producers in our region. Since 2021, an emergence of patients infected by CPE co-harbouring KPC and OXA-48-like carbapenemase has been observed (Figure S1). In particular, during the third and fourth wave of the COVID-19 pandemic (i.e. between January and December 2021) we observed a total of 60 patients infected by *K. pneumoniae* co-producing KPC and OXA-48-like carbapenemase.

In order to characterize the *K. pneumoniae* with double carbapenemase production and to evaluate the clonal relationships among them, we selected 10 clinical strains isolated from patients hospitalized in three different hospitals. AST revealed that *K. pneumoniae* isolates co-producing KPC and OXA-181 carbapenemase were resistant to meropenem/vaborbactam (median MIC 32 mg/L, IQR 16–32) and imipenem/relebactam (median MIC 4 mg/L, IQR 4–4), while 7 out of 10 of isolates (70%) were resistant to ceftazidime/avibactam (median MIC 24 mg/L, IQR 16–256). At the same time, 8 out of 10 of isolates (80%) resulted resistant to cefiderocol (median MIC of 16 mg/L, IQR 16–16) (Table 1). Of note, 6 out of 7 (85.7%) of ceftazidime/avibactam-resistant *K. pneumoniae* showed cross-resistance to cefiderocol (median MIC 16 mg/L, IQR 16–16), thus demonstrating a significant correlation between the mutations within the KPC gene and porin disruption at the basis of such resistance.

**Table 1.** Phenotypic characteristics of KPC- and OXA-181-co-producing *K. pneumoniae* strains included in this study

Isolate	KPC variant	Isolation source	Hospital	ICU recovered	MIC (mg/L)			
					CAZ/AVI	MEM/VAB	IPM/REL	FDC
BAT146	KPC-3	Blood	A	Yes	4	32	4	2
BO714	KPC-125	Bronchial aspirate	B	No	>256	16	4	16
BO739	KPC-3	Urine	B	No	8	32	4	16
BO743	KPC-121	Venous catheter	A	Yes	>256	32	8	16
BO761	KPC-3	Bronchial aspirate	B	Yes	16	16	8	1
BO793	KPC-66	Urine	C	No	16	32	4	8
BO830	KPC-68	Necrotic pancreatic tissue	B	Yes	64	64	4	16
BO837	KPC-3	Urine	C	No	4	32	4	16
BO999	KPC-31	Blood	B	Yes	>256	32	4	16
CAZ154	KPC-66	Blood	A	Yes	32	16	4	32

Reduced susceptibility to antimicrobial molecules is indicated in bold. CAZ/AVI, ceftazidime/avibactam; MEM/VAB, meropenem/vaborbactam; IPM/REL, imipenem/relebactam; FDC, cefiderocol.

Genome analysis revealed that all strains belonged to ST512 and exhibited a common genetic background correlated with resistance to various antimicrobial molecules including β-lactams aminoglycosides, colistin, fosfomycin, macrolides, phenicols, quaternary ammonium compounds, quinolones, rifamycin, sulphonamides, tetracyclines and trimethoprim (Table S1).

Analysis of carbapenemase genes revealed that 4 out of 10 *K. pneumoniae* strains harboured *bla*_KPC-3_, while 6 carried different *bla*_KPC_ variants including *bla*_KPC-66_, *bla*_KPC-68_, *bla*_KPC-31_, *bla*_KPC-121_ and *bla*_KPC-125_ (Figure S2). Of note, genome comparison among KPC and OXA-181 co-producers showed that clinical isolates carrying mutated *bla*_KPC-3_ exhibited higher MICs of cefiderocol (median MIC 16 mg/L, IQR 16–16) and ceftazidime/avibactam (median MIC 32 mg/L, IQR 32–256) than isolates carrying WT *bla*_KPC-3_ (cefiderocol median MIC 9 mg/L, IQR 1.5–16; ceftazidime/avibactam median MIC 6 mg/L, IQR 4–12). Also, analysis of the porin-encoding genes showed that all isolates carried a truncated OmpK35 at amino acid position 41, and glycine and aspartic acid insertion at position 135 within OmpK36 (Table S1).

Plasmid content analysis showed that all isolates harboured replicons belonging to the incompatibility types ColKP3, IncA/C2, IncFIB(K), IncFIB(pQil), IncFII(K) and IncX3, except for strain BO793 lacking the IncFIB(pQil) replicon (Table S1).

**Figure 1.**
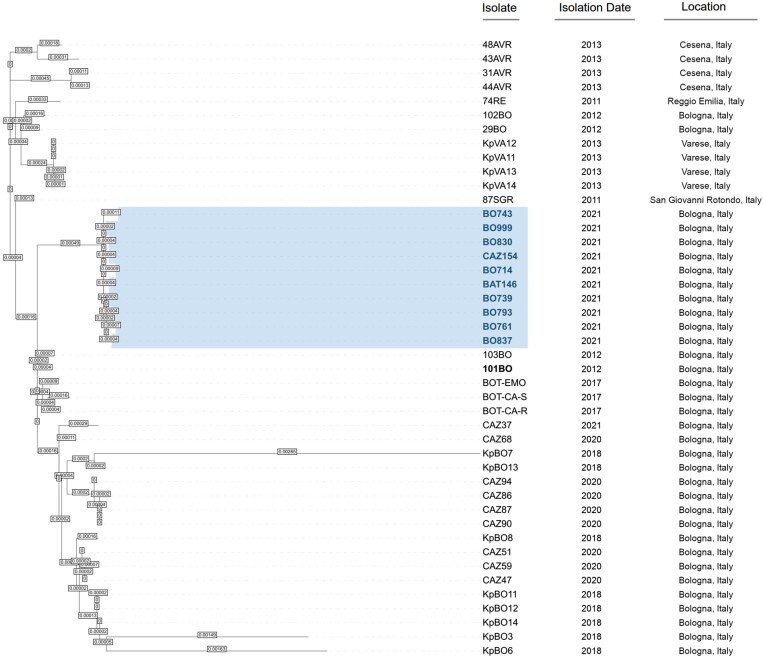
Phylogenetic tree based on core-genome SNPs of *K. pneumoniae* strains isolated in Northern Italy between 2011 and 2021. Strains presented in this study are highlighted in blue. Isolate 101BO was used as reference.

Phylogenetic analysis of KPC- and OXA-181-co-producing *K. pneumoniae* strains compared with strains harbouring different KPC variants isolated in Italy revealed that all isolates included in this study are strictly related and belong to the same monophyletic group (Figure 1).

## Discussion

During the COVID-19 pandemic different health-related strategies have been applied to counteract the global diffusion of SARS-CoV-2 virus, which have impacted on the patient population in different ways, including the widespread diffusion of antimicrobial resistance.^17,18^ In this context, we describe the changing epidemiology of CPE in the metropolitan area of Bologna (Northern part of Italy) prior to and during the COVID-19 pandemic. Our results show that during 2021 widespread emergence of CPE co-producing KPC and OXA-48-like carbapenemase was observed in the metropolitan area of Bologna, an endemic area for KPC producers. Of note, *K. pneumoniae* strains co-producing KPC and OXA-181 were resistant to the novel βL-βLICS, including ceftazidime/avibactam, meropenem/vaborbactam, imipenem/relebactam, and cefiderocol, thus limiting the antimicrobial options available for clinicians.

Our findings suggest that the resistance to meropenem/vaborbactam and imipenem/relebactam has been associated with the production of OXA-181 carbapenemase, which is unaffected by vaborbactam and relebactam inhibitors, while resistance to ceftazidime/avibactam has probably been associated with mutations within the KPC-3 carbapenemase due to the structural modifications occurring in the Ω-loop.^5,15^ In addition, all KPC-producing *K. pneumoniae* isolates included in this study showed high MICs of cefiderocol, thus indicating that the co-production of KPC and OXA-181 carbapenemase and disruption of OmpK35 and mutated OmpK36 (i.e. GD insertion at aa 134–135) porins associated with the co-production of different antimicrobial resistance determinants to β-lactams (i.e. mutations within the Ω-loop of carbapenemase genes such as *bla*_KPC-31_, *bla*_KPC-66_, *bla*_KPC-68_ and *bla*_KPC-121_) played a key role in the resistance activity against this molecule. At the same time, our results showed that all isolates harbouring KPC variants exhibited higher MICs of cefiderocol than WT KPC-3, indicating that structural modifications within this domain of the KPC enzyme are effective in conferring resistance to ceftazidime/avibactam and determine an increase in MICs of cefiderocol. These results are in accordance with previous studies, which demonstrated that in KPC producers different mutations within the KPC Ω-loop involved in resistance to ceftazidime/avibactam also impact significantly on the activity of cefiderocol.^11,12,19^

Although clinical *K. pneumoniae* strains co-producing KPC and OXA-181 were collected from different hospitals, phylogenetic analysis showed that all *K. pneumoniae* strains were closely related and segregated into a monophyletic group, suggesting widespread dissemination of a clonal strain during the third and fourth waves of the COVID-19 pandemic in this area.

In conclusion, our findings describe the widespread emergence of *K. pneumoniae* co-producing KPC and OXA-181 carbapenemase, which exhibited a pandrug phenotype associated with resistance to the novel molecules against MDR Gram-negative bacteria. Although βL-βLICs and cefiderocol should be used as a valid therapeutic alternative in difficult-to-treat (DTR) infections due to MDR pathogens in patients with limited therapeutic options, reasonable and controlled use of these antimicrobial molecules is fundamental in order to preserve the efficacy of novel molecules and to avoid the emergence and diffusion of resistance mechanisms in Gram-negative bacteria.

## Supplementary Material

dlad099_Supplementary_Data

## Supplementary data

Figures S1 and S2 and Table S1 are available as Supplementary data at *JAC-AMR* Online.
