# Supplementary material for: Clonal dissemination of Klebsiella pneumoniae resistant to cefiderocol, ceftazidime/avibactam, meropenem/vaborbactam and imipenem/relebactam co-producing KPC and OXA-181 carbapenemase
Source: JAC Antimicrob Resist. 2023 Aug 17;5(4):dlad099. doi: 10.1093/jacamr/dlad099 (PMC10433922; doi:10.1093/jacamr/dlad099)

**Table S1**. Genotypic characteristics of KPC and OXA-181 co-producing *Klebsiella pneumoniae* strains included in this study

| **Isolate** | **Accession number** | **MLST** | **Genetic determinants** | | | | **Porins** | |  |
| --- | --- | --- | --- | --- | --- | --- | --- | --- | --- |
|  |  |  | ***bla*_KPC_** | ***bla*_OXA-181_** | **β-lactamase** |  | ***ompk*35** | ***ompK*36** | **Plasmid replicons** |
| BAT146 | SAMN27122169 | 512 | *bla*_KPC-3_ | *bla*_OXA-181_ | *bla*_TEM-1,_ *bla*_SHV-11,_ *bla*_CMY-16,_ *bla*_OXA-10_ |  | Truncated at aa 41 | GD134–135  insertion | *ColKP3 IncC IncFIB(K) IncFIB(pQil) IncFII(K) IncX3* |
| BO714 | SAMN27594434 | 512 | *bla*_KPC-125_ | *bla*_OXA-181_ | *bla*_TEM-1,_ *bla*_SHV-11,_ *bla*_CMY-16,_ *bla*_OXA-10_ |  | Truncated at aa 41 | GD134–135  insertion | *ColKP3 IncC IncFIB(K) IncFIB(pQil) IncFII(K) IncX3* |
| BO739 | SAMN27122162 | 512 | *bla*_KPC-3_ | *bla*_OXA-181_ | *bla*_TEM-1,_ *bla*_SHV-11,_ *bla*_CMY-16,_ *bla*_OXA-10,_ |  | Truncated at aa 41 | GD134–135  insertion | *ColKP3 IncC IncFIB(K) IncFIB(pQil) IncFII(K) IncX3* |
| BO743 | SAMN27596901 | 512 | *bla*_KPC-121_ | *bla*_OXA-181_ | *bla*_TEM-1,_ *bla*_SHV-11,_ *bla*_CMY-16,_ *bla*_OXA-10_ |  | Truncated at aa 41 | GD134–135  insertion | *ColKP3 IncC IncFIB(K) IncFIB(pQil) IncFII(K) IncX3* |
| BO761 | SAMN27122167 | 512 | *bla*_KPC-3_ | *bla*_OXA-181_ | *bla*_TEM-1,_ *bla*_SHV-11,_ *bla*_CMY-16_ |  | Truncated at aa 41 | GD134–135  insertion | *ColKP3 IncC IncFIB(K) IncFIB(pQil) IncFII(K) IncX3* |
| BO793 | SAMN27122160 | 512 | *bla*_KPC-66_ | *bla*_OXA-181_ | *bla*_TEM-1,_ *bla*_SHV-11,_ *bla*_CMY-16_ |  | Truncated at aa 41 | GD134–135  insertion | *ColKP3 IncC IncFIB(K) IncFII(K) IncX3* |
| BO830 | SAMN27122159 | 512 | *bla*_KPC-68_ | *bla*_OXA181_ | *bla*_TEM-1,_ *bla*_SHV-11,_*bla*_CMY-16 ,_ *bla*_OXA-10_ |  | Truncated at aa 41 | GD134–135  insertion | *ColKP3 IncC IncFIB(K) IncFIB(pQil) IncFII(K) IncX3* |
| BO837 | SAMN27122165 | 512 | *bla*_KPC-3_ | *bla*_OXA-181_ | *bla*_TEM-1,_ *bla*_SHV-11,_ *bla*_CMY-16,_ *bla*_OXA-10_ |  | Truncated at aa 41 | GD134–135  insertion | *ColKP3 IncC IncFIB(K) IncFIB(pQil) IncFII(K) IncX3* |
| BO999 | SAMN27122166 | 512 | *bla*_KPC-31_ | *bla*_OXA-181_ | *bla*_TEM-1,_ *bla*_SHV-11,_ *bla*_CMY-16,_ *bla*_OXA-10_ |  | Truncated at aa 41 | GD134–135  insertion | *ColKP3 IncC IncFIB(K) IncFIB(pQil) IncFII(K) IncX3* |
| CAZ154 | SAMN31158792 | 512 | *bla*_KPC-66_ | *bla*_OXA-181_ | *bla*_TEM-1,_ *bla*_SHV-11,_ *bla*_CMY-16 ,_ *bla_O_*_XA-10_ |  | Truncated at aa 41 | GD134–135  insertion | *ColKP3 IncC IncFIB(K) IncFIB(pQil) IncFII(K) IncX3* |

**Figure S1.** Distribution of carbapenemase type on clinical samples between January 2019 and December 2021


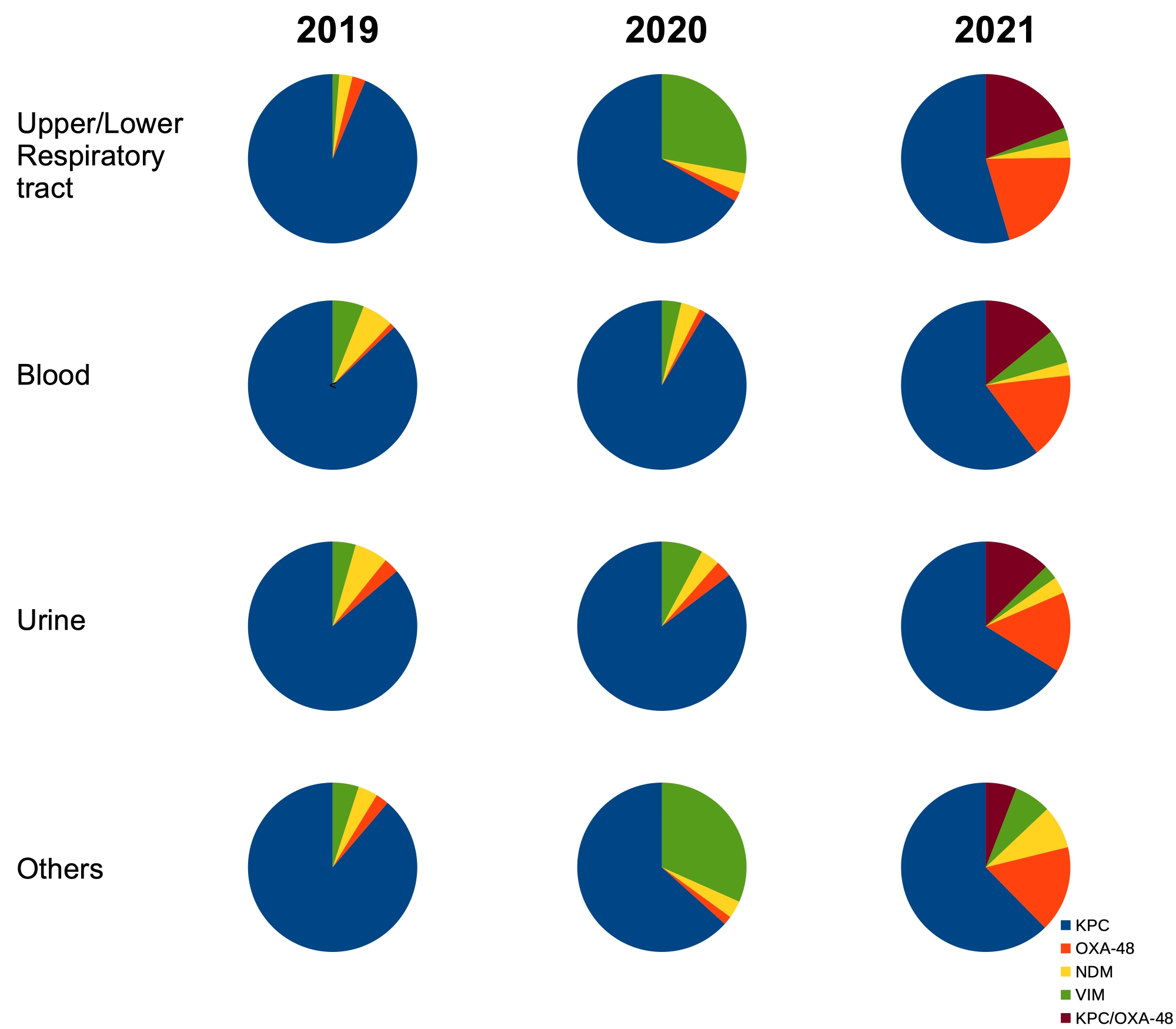


**Figure S2**. Amino acid alignment of KPC variants. The Ω-loop is highlighted in yellow.


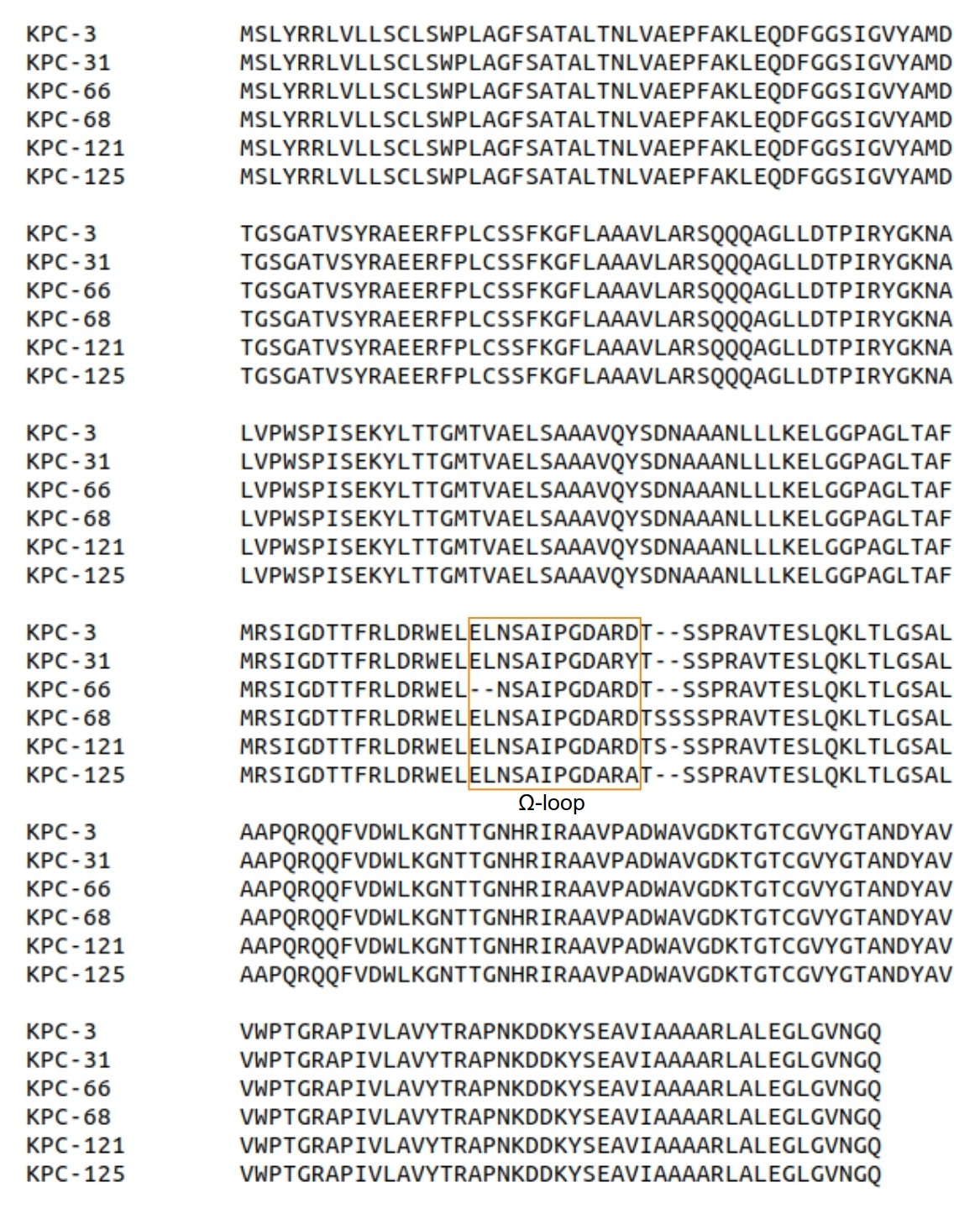

Supplement: dlad099_Supplementary_Data [file dlad099_supplementary_data.docx]
